# Case Report: Catatonic Stupor in Behavioral Variant Frontotemporal Dementia

**DOI:** 10.3389/fneur.2021.798264

**Published:** 2022-01-18

**Authors:** Gustavo Campos de França, Henrique Carneiro de Barros Barreto, Thiago Paranhos, Julio Cesar Nunes, Ricardo de Oliveira-Souza

**Affiliations:** ^1^The D'Or Institute for Research and Education, Rio de Janeiro, Brazil; ^2^The Federal University of the State of Rio de Janeiro, Gaffrée e Guinle Hospital, Rio de Janeiro, Brazil; ^3^The Federal University of Rio de Janeiro, Clementino Fraga Filho Hospital, Rio de Janeiro, Brazil

**Keywords:** abulia minor, akinetic mutism, catatonia, catatonic stupor, frontotemporal dementia (FTD), hypersexuality, Pick's disease

## Abstract

Catatonia is a psychomotor syndrome common to several medical and neuropsychiatric disorders. Here, we report on the case of a 95-year-old woman who underwent a radical change in personality characterized by sexual disinhibition, and physical and verbal aggressiveness. Over several months, she developed verbal stereotypies, gait deterioration, and double incontinence. She eventually developed mutism and an active opposition to all attempts to be fed or cared for. Benzodiazepines, olanzapine and electroconvulsive therapy were of no benefit. Magnetic resonance imaging revealed asymmetric (more severe on the right) frontotemporal, parietal, and upper brainstem atrophy. She died from sepsis without recovering from stupor seven years after the onset of symptoms. We believe that the initial behavioral disinhibition was related to the frontotemporal injury, whereas catatonic stupor reflected the progression of the degenerative process to the parietal cortices. Our case adds to the small number of cases of catatonia as a symptom of degenerative dementia. It also supports the idea that damage to the parietal cortex gives rise to pathological avoidance of which catatonic stupor represents an extreme form.

## Introduction

Originally conceived as a cyclical illness that unfolded along a predictable course that usually ended in dementia (1) or a subtype of schizophrenia (2), catatonia is currently regarded as a complex psychomotor syndrome common to several mental and physical disorders (3). As a symptom of cerebral and systemic disease, catatonia is as non-specific as, for example, delirium or coma (4). For the purposes of the present communication, we use “catatonic stupor” to refer to a particular symptom of catatonia in which all sorts of interaction with the environment are abolished or significantly diminished; this critically includes interpersonal communication by means of gesture, writing and speech (mutism). According to this view, a patient who is mute but communicates his intentions and understanding through gesturing may be catatonic, but not in catatonic stupor (5, 6). In contrast to “neurological” stupor (7), catatonic stupor is one of the most dramatic presentations of catatonia, in which patients lie mute, immobile, and unwilling to entertain meaningful interaction with the environment (5). At times, they adopt odd and uncomfortable postures, their bodies being animated only by stereotypies of head, gaze, mouth, and hands (8). Catatonic stupor is an extreme form of negativism, which encompasses a host of symptoms that reflect an active or passive refusal to interact with people and objects (9). Like catatonia in general, catatonic stupor is a non-specific syndrome common to a variety of systemic and neuropsychiatric diseases whose prompt recognition may be lifesaving (10). Neurologists have long known that catatonia may be the chief or sole manifestation of brain disease (11). However, the first unquestionable evidence for a causal link between catatonia and brain damage was provided by cases of lethargic encephalitis, which raged in the Northern Hemisphere in the first quarter of the twentieth century (12). Interest in this association gained new impetus following the recognition that most classical psychopathological syndromes, such as catatonic stupor, may be replicated by certain forms of frontotemporal dementia (FTD), especially in their early stages (13–15).

Research on the prevalence of catatonia in the elderly has met with conflicting results. Part of the discrepancies are probably due to differences in the diagnostic composition of the samples of patients studied and the methods of study. For example, catatonia was diagnosed in 40% of the 106 patients admitted to an acute geriatric psychiatric ward. The authors noticed a strong reciprocal association between depression and catatonia in their sample (16). In contrast, in 98 patients over 65 years of age admitted to the psychiatric ward of a general hospital, Takács et al. (17) found a prevalence of catatonia in 17% of the cases, most of which were due to a general medical condition. Because catatonic symptoms are commonly found in certain types of primary neurodegenerative dementias [e.g., Lakshmana et al. (18); Ishmora et al. (19)], it is surprising that dementia was not particularly associated with catatonia in either study. The reasons for this are unclear, but physicians' lack of familiarity with the manifestations of catatonia may be a sufficient explanation in most cases.

The goal of this communication is to present the case of an elderly woman who underwent a radical change in personality that evolved into a catatonic stupor from which she would never recover. Written informed consent was obtained from her only daughter for the publication of the case as it is.

## Case Presentation

A 95-year-old right-handed housewife, native Portuguese speaker, without a premorbid history of neurological or psychiatric illnesses and 5 years of formal education, was brought by her daughter with the eyes shut and unresponsive to all sorts of verbal and gestural attempts to elicit any kind of intelligible response. At the age of eighty-nine, the patient became afraid of being robbed and assaulted, especially at home during the night. Her daughter and son-in-law, with whom she had lived for many years, did not at first give much importance to the fact; they assumed that, after all, their neighborhood was one of the areas in the city in which drug trafficking was a reason for major public concern. In their view, the patient was understandably apprehensive. However, they did not fail to realize that the patient, a model of moral strength, was unusually afraid of a situation that had never intimidated her before. No matter how hard her daughter assured her or attempted to convince her of how safe they were at their seventh-floor apartment, the patient's fears increased and became especially troublesome when she started coming to their bed at dawn without warning. A few months after unsuccessful attempts to calm her down, her daughter took her to a psychiatrist. She received a diagnosis of major depression and was prescribed 50 mg of sertraline at bedtime. She improved in a few weeks and did not bother her daughter at night for a while. However, a few months later, they noted that she was less active and socially withdrawn. Over time, she would react emotionally only to her little great-grandson whom she had always loved. Although she stayed up all day long, she spoke only when people addressed her directly or when she wanted to drink or eat. The daily dose of sertraline was increased to 100 mg without benefit. The psychiatrist then entertained the possibility of SSRI-related apathy, and gradually suspended the drug. As the apathy did not improve, she was prescribed a maintenance dose of 50 mg of sertraline. Because psychomotor agitation did not recur, further changes in medication were not done.

Three years later, her relatives noticed that the patient's lifelong stern and moralist, albeit gentle, character was undergoing a remarkable change. Her language became “liberal,” and she would insult anyone who attempted to take care of her. At the same time, she became “unusually kind” to her grandsons as well as to her granddaughters' boyfriends. She confided to a close friend of her daughter that she was engaged to a married man, but did not want her family to know about the affair before he divorced his wife. When she had to go out for some reason, she insisted to sit in the front seat by the cab driver. On these occasions, her daughter, who sat in the back seat, had to keep a constant watch on her, because the patient would surreptitiously try to touch the driver's private parts. She compulsively engaged in genital self-stimulation and attained several orgasms per day accompanied by loud moans, unconcerned about being observed. Although her food preferences did not change, her refusal to eat incurred in considerable loss of weight. A year before her first appointment with us, she began speaking a strange language, “as if she were ‘speaking in tongues'.” Over the ensuing months, her gait deteriorated, and she eventually became unable to walk and doubly incontinent.

In the few weeks that antedated our first appointment, she became mute and would rarely open her eyes. By then, she would spend most of the time laying over the left side of the body embracing her flexed legs in a genuflected attitude. She would not ask for water, food, or grooming; on the contrary, she stubbornly refused any kind of contact, contracting her lips and slapping anyone who offered her food and water.

**Figure 1:**
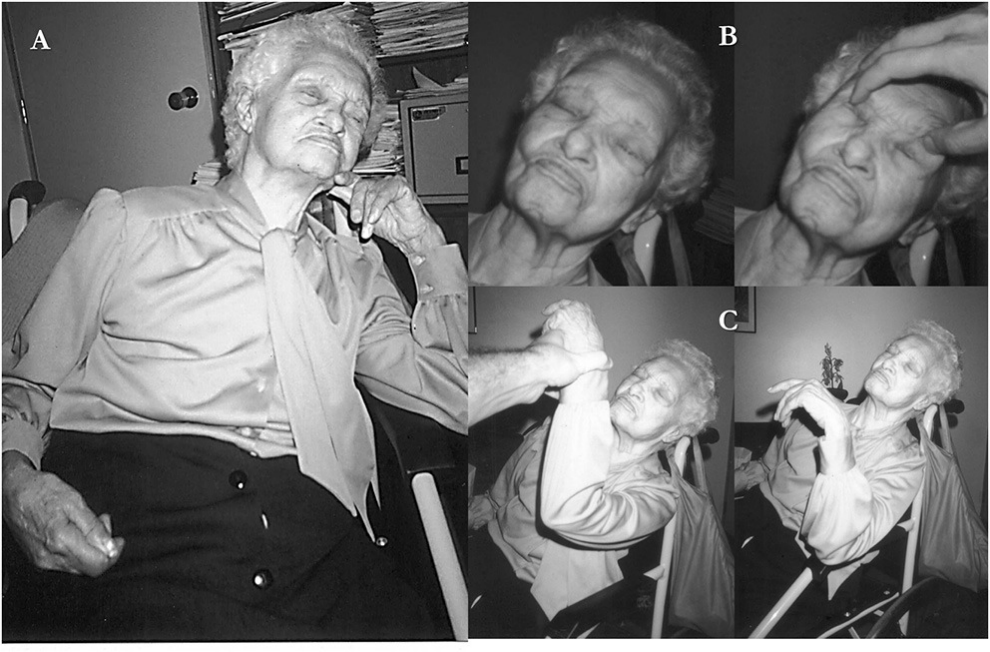
Persistent catatonic features of patient. **(A)** This spontaneously adopted attitude could remain unchanged for hours on end (catalepsy). **(B)** Active opposition to passive elevation of the lids (active negativism). **(C)** Sustained attitude of the left arm which was passively imposed by examiner (waxy flexibility).

**Figure 2:**
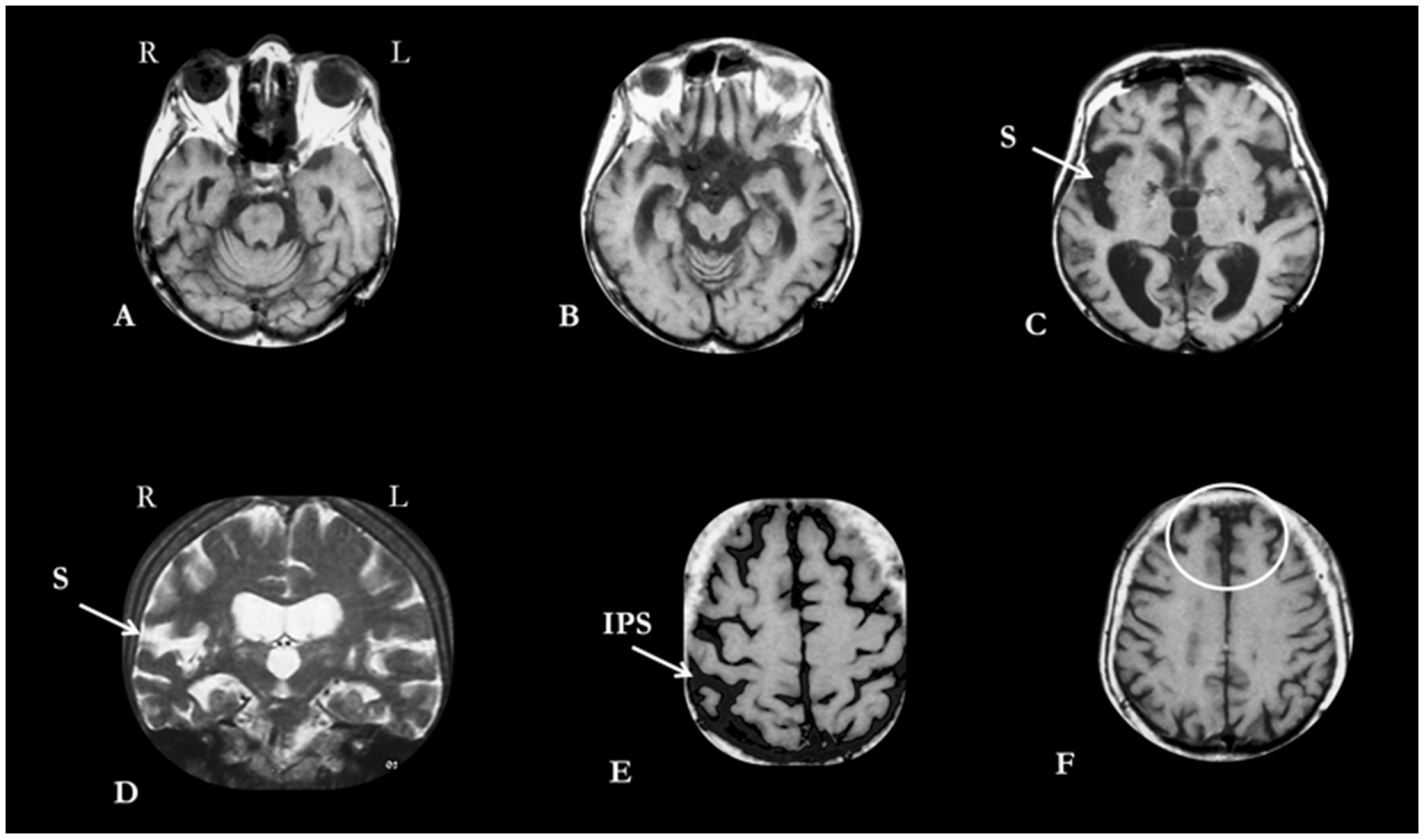
MRI of patient obtained during the stage of catatonic stupor. **(A,B)** Vermal atrophy, and volumetric reduction of right side of ventral pons **(A)** and cerebral peduncle **(B)**. **(C,D)** Enlargement of sylvian fissures (S), more pronounced on the right, is evident on both axial **(C)** and coronal **(D)** planes. **(E)** Enlargement of intraparietal sulci (IPS), more pronounced on the right. **(F)** More evident atrophy of polar and superomedial prefrontal lobes (circled in white) in relation to the posteromedial parietal lobes. L, left; R, right.

**Figure 3:**
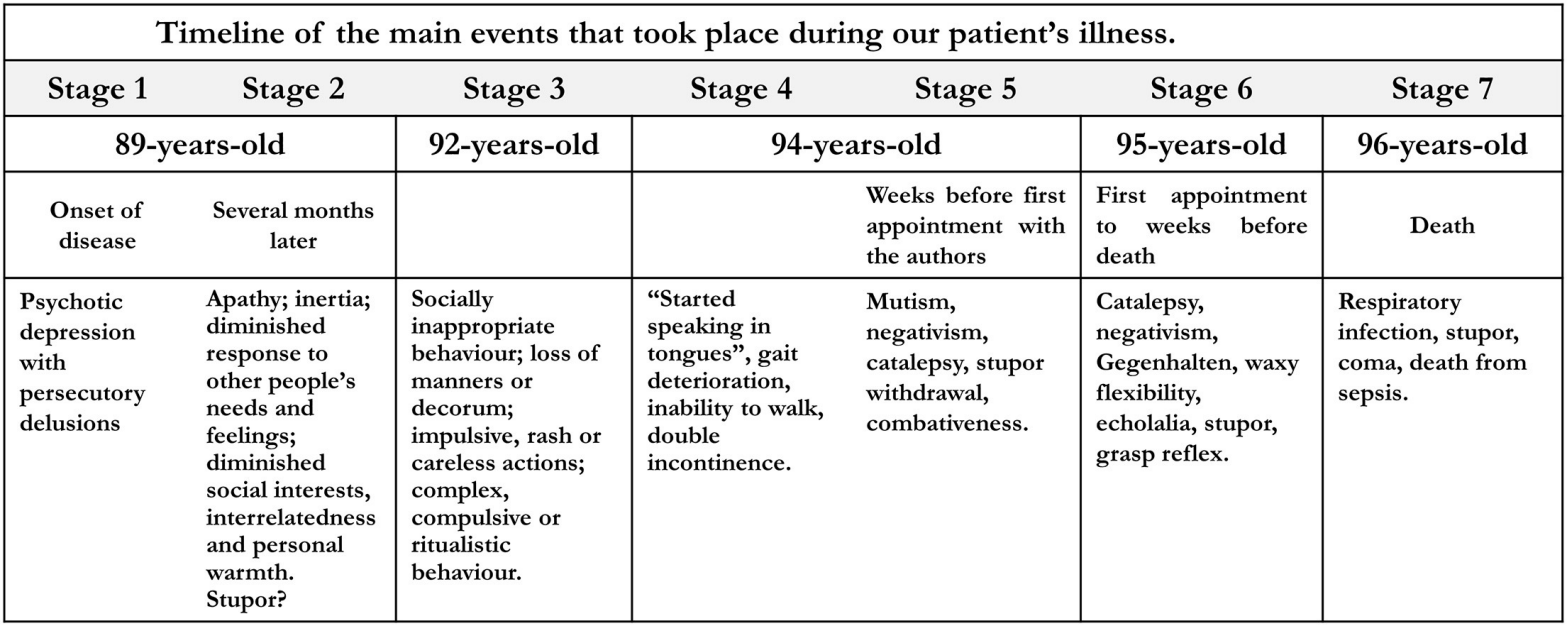
Timeline of the main events that took place during our patient's illness.

On examination, she kept her eyes shut most of the time (Figure 1A), vigorously resisting even gentle attempts by the examiner to open them (Figure 1B); she sustained passively imposed attitudes of body segments for several minutes (Figure 1C). Sometimes, she opened the eyes and answered simple questions with unintelligible words (for example, “Sabagaah” to “what day of the week is today?”). At times, she echoed the examiner's speech, (“1, 2, 3…”). Oral prehension and right manual grasp reflexes were easily elicited by stroking her lips and palm, respectively. At the time, she met 12 of 14 screening items and scored 41 of 69 points on the Bush-Francis Catatonia Rating Scale (Supplementary Table 1A). On the Northoff Catatonia Scale, she obtained a total of 34 points (Motor subscale = 10; Affective subscale = 6, Behavioral subscale = 18). A challenge of 10 mg diazepam IV produced no effect. Olanzapine, 10 mg of at bedtime, was prescribed for 6 weeks with no benefit, and was suspended. Likewise, six sessions of electroconvulsive therapy failed to produce any benefit and were discontinued. Electroconvulsive therapy was administered three times per week with a square-wave, brief-pulse, constant-current device. After the patient was sedated with a short-acting anesthetic and curarized, a 5-sec electrical stimulus was applied simultaneously to each temple. Following the end of stimulation, a convulsive seizure lasting 20–30 secs was seen in the forearm using the pressure-cuff technique. No benefit was noted after 6 sessions, and the treatment was also discontinued. Magnetic resonance imaging (MRI) revealed asymmetric cerebrocerebellar and brainstem atrophy, which was more marked in the right frontal and parietal lobes, right ventral midbrain and pons as well as in the anterior temporal lobes (Figure 2). An abnormally high caudate index (= 0.19; normal ≤ 0.09) and a normal bifrontal index (0.37; normal ≥ 0.30) indicated caudate atrophy greater than expected from hemispheric atrophy alone (20). A diagnosis of behavioral variant frontotemporal dementia (bvFTD) was made (Supplementary Table 1B). A comprehensive battery of laboratory tests that included blood cell counts, ESR, glucose, BUN, serum lipid screening, thyroid hormones and TSH, PTH, hepatic function tests, VDRL and FTA-ABS, and B and D vitamins were negative or within normal limits. Over the ensuing years, the patient was treated conservatively at home, being fed through gastrostomy. Given the lack of response to several medications, no other attempts to treat catatonia were made. In her last days she developed an acute respiratory infection that prompted her admission to a public hospital where she stayed for a few days. Shortly before her death, she fell into a coma due to sepsis. She died at age 96, seven years after the first symptoms of the disease (Figure 3).

## Discussion

Our patient developed an insidious change in personality characterized by sexual disinhibition, and verbal and physical aggressiveness against anyone who attempted to feed and groom her. Her speech became increasingly non-sensical until her verbal output became restricted to a few words, echolalia, and interjections. Her emotions became coarse, and she lost her usual social graces and manners. Lacking insight into these changes, her behavior became quite troublesome for caregivers and a source of embarrassment for her relatives. During the early stages of the disease, her bizarre social behavior contrasted with a relative preservation of visuospatial orientation and recognition of friends and relatives. She eventually progressed into a catatonic stupor from which she never recovered until she died. MRI showed predominant atrophy of the frontobasal and anterior temporal lobes as well as of the cerebral peduncle and basis pontis, all of which were more pronounced on the right; the ventral brainstem atrophy was probably due to secondary degeneration of the frontotemporopontine tracts (21). Caudate atrophy, in contrast, was fairly symmetric (Figure 2). These findings were consistent with a clinicoanatomical diagnosis of probable bvFTD (Table 1). The diagnosis of bvFTD was further supported by the asymmetrical hemispheric atrophy, which is typical of the frontotemporal degenerations (36). The age of our patient differs from that of most FTD cases, which usually manifest much earlier. However, this should not be a deterrent to diagnosis as there are well-documented cases of FTD beginning after 65 years of age.

**Table 1:** Catatonia in frontotemporal atrophy (cases of catatonic stupor highlighted in gray).

**References**	**Year**	**Id**	**Sex**	**Age at onset** **(in years)**	**Occupation**	**Main clinical findings**	**Anatomical findings**	**Premorbid history**	**Family history**
Ruff and Russakoff (22)	1980	Mrs. A	F	53	NR	Marked deterioration in personal hygiene and housekeeping skills, sleep irregularities, decision-making difficulties, and depressed affect. A diagnosis of psychotic depression was made, and she was started on chlorpromazine with temporary improvement in affect and personal hygiene. A year later, she was hospitalized for mutism, social withdrawal, apathy, sadness, psychomotor slowness, and loss of insight. A diagnosis of FTD was made, but this was dismissed as she fully recovered with a regular therapeutic scheme with typical antipsychotics.	Bilateral frontal sharp waves and excess theta activity (EEG). Bilateral frontal lobe atrophy without ventricular enlargement (CT).	Arterial hypertension.	NR
Muñoz-Garcia and Ludwin (23)	1984	Patient 3	F	74	NR	Gradual mental deterioration over several years. She became catatonic, unresponsive, and unable to recognize others. Bilateral grasp reflexes and upgoing toes were present, but no other neurological signs were found. She developed peritonitis and septicemia, which led to death.	Marked frontotemporal and anterior cingulate atrophy with sparing of occipital lobes; atrophic cortex showed 90% loss of neurons, fibrillary astrocytosis, increased microglial cells, and loss of fibers. Large neuronal cytoplasmic inclusions, conforming to the classic description of Pick bodies (histopathology examination).	NR	NR
García-Valls et al. (24)	2006	NR	M	42	NR	Behavioral change at the age of 30: became irritable and aggressive, followed by deficits in propositional language, irritable mood, mutism, verbigeration, negativism, and stereotypies. Cognitive impairment consistent with frontal lobe dementia eventually developed.	Frontal atrophy (TC and MRI) with frontal hypoperfusion (SPECT) and hypometabolism (PET).	NR	NR
Kagan et al. (25)	2007	NR	F	43	Worked as an aide in an elderly care facility and in a kindergarten class.	Social withdrawal, flattened affect, compulsive grooming, increase in appetite and binging on carbohydrate-rich food, memory decline, decreased speech, and echolalia over the preceding 2 years. Diagnosed with psychotic depression. No response to several antidepressants and ECT. Neuropsychological tests evinced impaired attention, concentration, verbal and narrative memory, and perseveration. Lorazepam failed to produce significant improvement. FTD was a presumptive diagnosis.	Diffuse cerebral atrophy (MRI).	NR	NR
Suzuki et al. (26)	2009	NR	M	48	NR	Grimacing, stereotyped behavior and speech, mannerisms, increased anger, inappropriate grooming, disregard for social obligations, depressed mood, anxiety, suicidal ideation and disturbances of recent memory. First diagnosed with FTD. Complete resolution of symptoms after ECT changed diagnosis to catatonia with underlying bipolar disorder.	Frontal lobe atrophy (MRI).	NR	NR
Lauterbach et al. (27)	2010	Mrs. X	F	70	NR	Lability ranging from inactivity to disinhibition and impulsivity, emotional blunting, loss of concern for the impact of her behavior on others, wandering, hyperorality, aspontaneous speech, loss of insight, decline in personal hygiene, hyperorality, catalepsy, and incontinence. Rigidity with moderate resistance and Gegenhalten. Finally diagnosed as FTD with catatonic features partially responsive to memantine.	Bilateral (slightly more pronounced on the L) hypometabolism most marked in anterior and medial frontal cortices extending into anterior cingulate, preserved subcortical metabolism consistent with FTD (^18^FDG-PET).	NR	NR
		Mrs. Y	F	62	Teacher	Progressive apathy and inability to manage her classroom, dysexecutive, poor attention and memory stooped posture, micrographia, compulsive cleaning behaviors and L hemi-parkinsonism. Treatment for Parkinson's disease unsuccessful. Could no longer work and retired. The patient developed emotional blunting, decline in personal hygiene, distractibility, behavioral impersistence, hyperorality, aspontaneous mutism, dysphagia, restless legs with periodic leg movements (responsive to pramipexole) and urinary incontinence. Echolalic and perseverative speech, catatonic posturing of the upper extremities especially on walking, and a manneristic gait. Diagnosed with FTD with catatonic syndrome partially responsive to amantadine and lorazepam.	Mild ventricular enlargement and scattered chronic white matter intensities without evidence of hipocampal atrophy (MRI). Symmetric frontal and L>R parietal hypometabolism with preserved temporal and occipital lobe and subcortical metabolism (^18^FDG-PET).	Hypercholesterolemia and osteoporosis.	NR
Smith et al. (28)	2012	NR	M	73	NR	3-month progression of diminished speech output, obsessive-compulsive symptoms, and behavioral outbursts. Catatonic symptoms did not respond to ECT and lorazepam.	Moderately-to-severely reduced uptake in the frontotemporal lobes (L>R) (^18^FDG-PET).	NR	NR
		NR	M	68	NR	5-year progression of aphasic dementia and behavioral dyscontrol. Spontaneous resolution with supportive measures.	Marked fronto-temporo-parietal hypometabolism (L>R) (^LL^FDG-PET).	NR	NR
		NR	M	65	NR	9-month history of progressive abulia, personality changes, and changes in emotional expression.	Bilateral temporal greater than generalized atrophy (MRI).	NR	NR
Utumi et al. (29)	2013	NR	M	60	NR	Insomnia, depressive mood, anxiety, appetite loss, irritability, talkativeness, and grandiosity. Treated several times without success for depression and bipolar disorder. MRI indicated FTD. One year later, developed akinetic mutism, immobility, stupor, catalepsy, negativism and impulsivity, excitement, perseveration, echolalia, and verbigeration. Symptoms resolved with lorazepam and fluvoxamine.	Frontal lobe atrophy (MRI).	NR	NR
Isomura et al. (19)	2013	NR	F	50	NR	Progressive apathy and difficulty in household work. Eighteen years later, catatonic features developed and FTD was suspected based on neuroimaging findings. Zolpidem resolved symptoms for a few hours, decreasing BFCRS score from 26 to 3, without side effects.	Bilateral frontal and temporal atrophy (MRI). Severe perfusion decrease in both high-frontal lobes, and mild decrease in both temporal lobes. After zolpidem administration, perfusion decreased in the brainstem and improved in the thalamus and L parieto-temporal lobe and L cerebellar cortex (SPECT).	NR	No family history of mental or neurologic diseases or hypoxic episodes.
Holm (30)	2014	NR	M	66	NR	Treated with citalopram following attempted suicide. Three months later, staring and disorganized behavior and speech followed by psychomotor agitation and violent behavior; finally, became mute and unresponsive. Lorazepam and ECT resolved catatonia and improved cognitive impairment; 6 months after discharge, developed anxiety and motor and verbal symptoms, which partially responded to ECT with persistence of verbigeration, mannerisms and cognitive impairment. A similar state developed 3 months later, and partially remitted with ECT, venlafaxine and lorazepam. Neuroimaging findings associated with C9ORF72 mutation led to the diagnosis of FTD.	Mild prefrontal and cerebellar atrophy (CT).	Lost his job after traumatic brain injury at the age of 40 that resulted in cognitive impairment.	Brother with bvFTD, mother with anatomo-pathological and clinical history of FTD.
Ducharme et al. (31)	2015	NR	M	55	Lawyer	Functional decline, cognitive impairment and executive dysfunction on neuropsychological tests led to the diagnosis of possible FTD. Developed catatonia after receiving haloperidol, improved with lorazepam. Periods of depression alternating with catatonia, was eventually hospitalized with confusion and aphasia. Motor perseveration, psychomotor slowness, paraphasia and fluency deficits led to the diagnosis of catatonia, as neuroimaging findings were inconsistent with FTD.	Non-progressive diffuse cerebral atrophy (MRI); patchy frontotemporoparietal hypometabolism (^18^FDG-PET).	Bipolar I disorder well controlled with lithium; acute lithium-induced glomerulopathy.	Bipolar mood disorder
Jaime-Albornoz et al. (32)	2015	NR	F	65	NR	Weight loss due to refusal to eat. Admitted to psychogeriatric ward with mutism, mannerisms, automatic obedience, Gegenhalten, echophenomena, staring and posturing. Reversal of catatonic stupor in one week with lorazepam and zolpidem. A diagnosis of bvFTD was made.	NR	NR	NR
		NR	M	67	NR	Admitted to psychogeriatric ward with mutism, echolalia, combativeness, mannerisms, staring, withdrawal, Gegenhalten and posturing. Remission of catatonic stupor in one week with lorazepam and valproic acid. A diagnosis of primary progressive aphasia was made.	NR	NR	NR
Watanabe et al. (33)	2017	NR	M	58	Office clerk	Stereotyped behavior and speech, staring, poor hygiene, echolalia, intermittent mutism, stupor, negativism and incoherence. He was treated as a case of catatonia associated with brief psychotic disorder. There were no hallucinations or delusions. Apathy and inertia persisted in spite of the improvement of catatonia after treatment with antipsychotics. He was diagnosed with FTD. In 2 years he became mute and died of pneumonia.	Marked atrophy of frontal and temporal lobes (MRI).	Pulmonary tuberculosis.	Elder brother with a diagnosis of schizophrenia.
Sayadnasiri and Rezvani (34)	2019	NR	F	65	NR	Avolition, poor grooming, circadian inversion, mutism, stupor, intermittent unmotivated anger outbursts, speech deterioration, gait disturbances (without parkinsonism) and staring. Patient was diagnosed with catatonia associated with FTD. Catatonia improved with lorazepam, speech symptoms with zolpidem. She remained stable even after discontinuing both substances due to sedative effects.	Frontal lobe atrophy (MRI).	Arterial hypertension	NR
Bretag-Norris et al. (13)	2019	NR	F	66	NR	Anhedonia, anorexia, weight loss, self-neglect, and abulia. No response to mirtazapine. Progressive change in personality with loss of warmth and empathy. Worsening in 6 months with poor grooming, mutism, negativism and mannerisms. A diagnosis of probable FTD was made.	Frontotemporal atrophy (MRI) and L frontotemporal hypoperfusion (SPECT).	NR	NR
	2019	NR	M	51	NR	Hospitalized after manually avulsing his scrotum and degloving his penile shaft. Posturing, waxy flexibility, grimacing and stereotypy were noted. His family described him as apathetic. No response to benzodiazepines, zolpidem, and antipsychotics. A diagnosis of probable FTD was made.	Severe frontotemporal atrophy (MRI).	NR	Family history of early onset dementias with significant neuro-psychiatric features.
Pompanin et al. (35)	2021	NR	M	63	NR	Psychomotor retardation leading to mutism, immobility, and generalized rigidity over a one year period. Remission of catatonic stupor and depression with antidepressants, but his memory and executive functions remained abnormal (MMSE = 21/30) Three years later, his cognitive impairment had progressed further (MMSE = 14/30). A diagnosis of bvFTD was made.	Hypometabolism in L prefrontal, temporal, and parietal cortex (18FDG-PET/MRI). Eight months later, the L hemisphere hypometabolism persisted, but metabolism returned to normal in all other cortical areas.	NR	NR
Present case	2022	PL	F	91	Retired	Suicide ideation, followed by aggressiveness and sexual disinhibition. Three years later, began to speak a “strange language” and developed catatonic stupor, double incontinence, and grasp and sucking reflexes. Olanzapine and ECT failed to produce benefits. Neuroimaging supported the clinical diagnosis of bvFTD. Steadily worsened and died of sepsis.	Asymmetric cerebrocerebellar and brainstem atrophy, more evident in R frontal, parietal, and anterior temporal lobes, and R ventral midbrain and pons (MRI).	Late-life psychotic depression preceding the behavioral changes.	NR

*BFCRS, Bush-Francis Catatonia Rating Scale; bvFTD, behavioral variant frontotemporal dementia; CT, computerized tomography; ECT, electroconvulsive therapy; F, female; ^18^FDG-PET, fluorodeoxyglucose positron emission tomography; FTD, frontotemporal dementia; Id, Identification; L, left; M, male; MRI, magnetic resonance imaging; NR, not reported; R, right; SPECT, single photon emission computerized tomography*.

### Catatonic Stupor in Frontotemporal Dementia

As a rule, catatonia should be suspected of in patients exhibiting odd, hyperactive, or akinetic behaviors (39). Diagnostic criteria and rating scales are useful guides for the recognition and rating of catatonic symptoms (40–42). Although catatonia has been described in a few instances of Alzheimer's (43) and Lewy body disease (44), most cases of catatonia in dementia have been reported in patients with FTD (Table 1). In fact, catatonic symptoms such as perseverative and stereotyped behavior, echolalia, and mutism are core features of current operational definitions of bvFTD (37, 38). It is thus somewhat surprising that catatonia has not been reported more often in FTD, and that catatonic stupor has been reported even less frequently. In all, we were able to retrieve 21 cases of catatonia as a symptom of FTD from the literature. As shown in Table 1, nearly half developed catatonic stupor at some point. Atrophy of the frontal lobes alone or associated with temporal atrophy was present in all cases. In nine patients, the anatomical evidence was supplemented by functional alterations in the atrophic regions, as shown by EEG (*N* = 1), SPECT (*N* = 2), or PET (*N* = 6).

Catatonia in general, and catatonic stupor in particular, may pose unanticipated diagnostic challenges in patients with dementia (25). For example, Suzuki et al. (26) reported on the case of a 51-year-old man with catatonia, functional decline, emotional blunting, craving for sweets, mania, and frontal lobe atrophy on MRI. Electroconvulsive treatment completely resolved all symptoms, including dementia. The initial diagnosis of FTD was eventually changed to bipolar disorder.

The possibility that catatonia may be more common in FTD than usually reported (27, 29) does not mean that FTD is the only possible diagnosis in elderly patients who are mute and unresponsive, for at least seven superficially similar conditions may be distinguished on phenomenological, pathophysiological, and neuropathological grounds (Supplementary Table 2). In our practice, the three conditions that have most often been a source of diagnostic dilemmas are the same that have also been thus reported in the literature, namely, coma (45), akinetic mutism (46), and “psychogenic” (47). The correct diagnosis of catatonia and catatonic stupor will expectedly increase as awareness of its key features also increases among generalists and specialists. Even a cursory look at the literature of recent years shows that this awareness is increasing rapidly and exponentially.

### The Neural Underpinnings of Catatonic Stupor

If, on the one hand, little can be said about the neural underpinnings of the catatonic stupor of our patient, on the other hand, this single case fits nicely into what is currently known on the subject. The strength invested to oppose external influences, a hallmark of catatonic stupor, points to the engagement of neural circuits whose complexity contrasts with the more fundamental sleep-wake rhythms and reflex eye movements that survive in unresponsive wakeful patients (48). Several years ago, Denny-Brown (49) postulated the existence of two neural systems that mediate approach-avoidance inclinations toward people, objects, and events. This author was one of the first to show that lesions of the frontal lobes may give rise to actions that vary in complexity from the simpler grasp reflex (50) to complex utilization behaviors (51), all of which point to an abnormally heightened inclination to approach people and objects; parietal lesions, in contrast, enhance avoidance and withdrawal from the environment (52). This functional reciprocity indicates that the frontal and parietal lobes promote avoidance and approach behaviors, respectively (53). An analogous mechanism may be at play at least in a few cases of catatonic stupor. This hypothesis is supported by clinicopathological associations between catatonia with or without stupor and bilateral (54, 55) or unilateral right (56, 57) parietal lesions, respectively.

These early interpretations of the neurological underpinnings of catatonic symptom complexes concur with modern neuroimaging findings, which have provided additional insights into the neural mechanisms at work. The premotor, cingulate motor, and supplementary motor areas may be a critical correlate of the akinetic, or retarded, psychomotor symptoms of catatonia (58). Dysfunction of intrinsic (i.e., postsynaptic) cortical neurons that express the GABA_A_ receptor in these regions may be the substrate of the well-known remission of catatonic stupor by benzodiazepines (59) and its prompt reversal by flumazenil (60). In line with the present case, there is evidence that among patients with schizophrenia spectrum disorder, catatonia correlates with reduced cortical volume in frontoparietal regions (61).

### Catatonic Stupor, Abulia, Akinetic Mutism, and the Unresponsive Wakefulness Syndrome (Formerly, Apallic-Vegetative State)

From a neurological perspective, catatonic stupor is more complex than abulia minor, akinetic mutism, or the unresponsive wakefulness syndrome, all of which are common end-stages of dementia from diverse etiologies (62). Although sharing many points in common, abulia and catatonia differ in critical ways. For example, many years ago, we reported on the case of a patient with a diagnosis of coma due to a spontaneous hemorrhage localized to Nauta's mesolimbic area (63). The patient lay immobile with the eyes shut without spontaneous speech or movement. Several days later, a Resident was shocked to see that he was awake and perfectly aware of his surroundings when he casually asked him to open the mouth. As it turned out, he was not able to open the eyes because the hemorrhage destroyed the oculomotor nuclei and fascicles. He was so abulic that he never attempted to initiate any kind of contact, not even to ask for help; in contrast to akinetic mutism, he answered by gestures whenever prompted; moreover, he showed no signs of catatonia. This case supports the view that abulia and catatonia differ in critical aspects: even though patients with catatonia are often abulic, not all abulic patients are catatonic. It also endorses the view that abulia is linked to destruction of the ascending projections from the ventral tegmental area (64), a hypothesis that has been partially verified in a recent MRI study that found that the mesencephalon is shrunken in catatonic patients with schizophrenia spectrum disorders (65).

The illness of our patient followed two stages that imperceptibly merged into each other, namely, the change in personality and the catatonic stupor. We suggest that these stages mirrored the progression of the underlying cerebral degeneration. Thus, during the early years, she became socially disinhibited and aggressive, and her speech became increasingly economic and meaningless. Along the same period, her gait deteriorated and she became incontinent. This stage was supposedly symptomatic of the frontotemporal degeneration proper (66). During the later stages, which lasted several months, she gradually entered a state of catatonic stupor that possibly corresponded to the progression of degeneration to the parietal lobes (67). Taken together, we believe that the progression of degeneration to the parietal lobes overcame the initial disinhibitory effects of the earlier frontotemporal injury and eventually produced a net imbalance toward the avoidance pole of the approach-avoidance continuum (68), of which catatonic stupor represents the maximum behavioral expression.

## Conclusion

Catatonic stupor in the elderly may be the sole or main symptom of degenerative dementia, especially FTD. The diagnosis in such cases may be delayed when it falls into this no man's land that neurologists and psychiatrists believe to be the province of the other. The scarcity of practitioners who are skilled at both neurology and psychiatry may unwittingly relegate many patients to a sort of “specialties limbo.” Clearly, there is an urgent need to revive the *practice* of neuropsychiatry in the light of the technological achievements that have allowed the *in vivo* study of the neurological underpinnings of normal and abnormal human mind and behavior (69).

## Data Availability Statement

The original contributions presented in the study are included in the article/Supplementary Material, further inquiries can be directed to the corresponding author.

## Ethics Statement

Written informed consent was obtained from the individual's legal guardian/next of kin for the publication of any potentially identifiable images or data included in this article.

## Author Contributions

All authors have contributed to the discussion and writing processes of this paper.

## Conflict of Interest

The authors declare that the research was conducted in the absence of any commercial or financial relationships that could be construed as a potential conflict of interest.

## Publisher's Note

All claims expressed in this article are solely those of the authors and do not necessarily represent those of their affiliated organizations, or those of the publisher, the editors and the reviewers. Any product that may be evaluated in this article, or claim that may be made by its manufacturer, is not guaranteed or endorsed by the publisher.
