# Supplementary material for: Case Report: Catatonic Stupor in Behavioral Variant Frontotemporal Dementia
Source: Front Neurol. 2022 Jan 18;12:798264. doi: 10.3389/fneur.2021.798264 (PMC8805594; doi:10.3389/fneur.2021.798264)
Supplement: Supplementary file 1 [file Table_1.docx]

| Supplementary Table 1.A. Patient Ratings on the Bush-Francis Catatonia Rating Scale* | | | | | | | | | | | | | | | |  |
| --- | --- | --- | --- | --- | --- | --- | --- | --- | --- | --- | --- | --- | --- | --- | --- | --- |
| Items | | | | ●/— | Score | |  | | | Items | | | ●/— | Score | |  |
|  | | | |  |  | |  | | |  | | |  |  | |  |
| 1 | | Excitement | | — | 0 | |  | | | 13 | Waxy flexibility | | ● | 3 | |  |
| 2 | | Immobility/stupor | | ● | 3 | |  | | | 14 | Withdrawal | | ● | 3 | |  |
| 3 | | Mutism | | ● | 3 | |  | | | 15 | Impulsivity | | NA | 0 | |  |
| 4 | | Staring | | ● | 3 | |  | | | 16 | Automatic obedience | | NA | 0 | |  |
| 5 | | Posturing/catalepsy | | ● | 2 | |  | | | 17 | Mitgehen | | NA | 3 | |  |
| 6 | | Grimacing | | ● | 3 | |  | | | 18 | Gegenhalten | | NA | 3 | |  |
| 7 | | Echopraxia/echolalia | | ● | 2 | |  | | | 19 | Ambitendency | | NA | 0 | |  |
| 8 | | Stereotypy | | ● | 2 | |  | | | 20 | Grasp reflex | | NA | 3 | |  |
| 9 | | Mannerisms | | — | 0 | |  | | | 21 | Perseveration | | NA | 0 | |  |
| 10 | | Verbigeration | | ● | 2 | |  | | | 22 | Combativeness | | NA | 2 | |  |
| 11 | | Rigidity | | ● | 2 | |  | | | 23 | Autonomic abnormality | | NA | 0 | |  |
| 12 | | Negativism | | ● | 2 | |  | | |  |  | |  |  | |  |
|  | | | | |  | |  | | |  | | | |  | |  |
| * Items 1-14 are screening items scored as present (●) or absent (—). NA: Not applicable. | | | | | | | | | | | | | | | |  |
|  | | | | | | | | | | | | | | | |  |
| Supplementary Table 1. B. Diagnosis of bvFTD according to the Neary *et al*. (1998) and the Rascovsky *et al*. (2011) criteria. A symbol (●) indicates that the feature was present, (—) indicates that the feature was absent, and (±) indicates that feature might have been present, but the evidence is weak. | | | | | | | | | | | | | | | | |
| Neary *et al*. (1998) | | | | | |  | |  | Rascovsky *et al*. (2011) | | | | | |  | |
| Clinical diagnostic features: Character change and disordered social conduct are the dominant features initially and throughout the disease course. Instrumental functions of perception, spatial skills, praxis, and memory are intact or relatively well preserved. | | | | | | ● | |  | **I. Neurodegenerative disease**: Progressive deterioration of behavior and/or cognition. | | | | | | ● | |
|  |  |  |  |  |  |  |  |  | **II. Possible bvFTD** | | | | | |  | |
|  |  |  |  |  |  |  |  |  | Three of the following behavioral/cognitive symptoms (A-F): | | | | | | | |
|  | | | | | |  | |  | A. At least one symptom of early behavioral disinhibition | | | | | | | |
| 1. Core diagnostic features | | | | | |  | |  |  | | | A.1. Socially inappropriate behavior | | | ● | |
| A. Insidious onset and gradual progression | | | | | | ● | |  |  | | | A.2. Loss of manners or decorum | | | ● | |
| B. Early decline in social interpersonal conduct | | | | | | ● | |  |  | | | A.3. Impulsive, rash or careless actions | | | ● | |
| C. Early impairment in regulation of personal conduct | | | | | | ● | |  | B. At least one symptom of early apathy or inertia | | | | | | | |
| D. Early emotional blunting | | | | | | ● | |  |  | | | B.1. Apathy | | | ● | |
| E. Early loss of insight | | | | | | ● | |  |  | | | B.2. Inertia | | | ● | |
| II. Supportive diagnostic features | | | | | |  | |  | C. At least one symptom of early loss of sympathy or empathy | | | | | | | |
| A. Behavioral disorder | | | | | |  | |  |  |  |  |  |  |  |  |  |
|  | 1. Decline in personal hygiene and grooming | | | | | ● | |  |  | | | C.1. Diminished response to other people’s needs and feelings | | | ● | |
|  | 2. Mental rigidity and inflexibility | | | | | ● | |  |  | | |  |  |  |  | |
|  | 3. Distractibility and impersistence | | | | | ● | |  |  | | | C.2. Diminished social interest, interrelated-ness or personal warmth | | | ● | |
|  | 4. Hyperorality and dietary changes | | | | | — | |  |  | | |  |  |  |  | |
|  | 5. Perseverative and stereotyped behavior | | | | | — | |  | D. At least one symptom of early perseverative, stereotyped or compulsive/ritualistic behavior | | | | | | | |
|  | 6. Utilization behavior | | | | | — | |  |  |  |  |  |  |  |  |  |
| B. Speech and language | | | | | |  | |  |  | | | D.1. Simple repetitive movements | | | ± | |
|  | 1. Altered speech output | | | | |  | |  |  | | | D.2. Complex, compulsive or ritualistic beha-viors | | | ● | |
|  |  | | a. Aspontaneity and economy of speech | | | ● | |  |  | | |  |  |  |  | |
|  |  | | b. Press of speech | | | — | |  |  | | | D.3. Stereotypy of speech | | | ● | |
|  | 2. Stereotypy of speech | | | | | ● | |  | E. At least one of the following symptoms of hyperorality and dietary changes: | | | | | | | |
|  | 3. Echolalia | | | | | — | |  |  |  |  |  |  |  |  |  |
|  | 4. Perseveration | | | | | — | |  |  | | | E.1. Altered food preferences | | | — | |
|  | 5. Mutism | | | | | ● | |  |  | | | E.2. Binge eating, increased consumption of al-cohol or cigarettes | | | — | |
| C. Physical signs | | | | | |  | |  |  | | |  |  |  |  | |
|  | 1. Primitive reflexes | | | | | ● | |  |  | | | E.3. Oral exploration or consumption of inedi- | | | — | |
|  | 2. Incontinence | | | | | ● | |  |  | | | ble objects | | |  | |
|  | 3. Akinesia, rigidity, and tremor | | | | | ● | |  | F. Neuropsychological profile: all of the following symp-toms: | | | | | | | |
|  | 4. Low and labile blood pressure | | | | | — | |  |  |  |  |  |  |  |  |  |
| D. Investigations | | | | | |  | |  |  | | | F.1. Deficits in executive tasks | | | ± | |
|  | 1. Neuropsychology: significant impairment on executive tests in the absence of severe amnesia, aphasia, or percepto-spatial disorder | | | | | ± | |  |  | | | F.2. Relative sparing of episodic memory | | | ● | |
|  |  |  |  |  |  |  |  |  |  | | | F.3. Relative sparing of visuospatial skills | | | ● | |
|  |  |  |  |  |  |  |  |  | **III. Probable bvFTD** | | | | | | | |
|  | 2. EEG: normal | | | | | — | |  | All of the following symptoms (A-C) | | | | | |  | |
|  | 3. Brain imaging: predominant frontal and/or ante-rior temporal abnormality | | | | | ● | |  | A. Meets criteria for possible bvFTD | | | | | | ● | |
|  |  |  |  |  |  |  |  |  | B. Exhibits significant functional decline | | | | | | ● | |
|  |  | | | | |  | |  | C. At least one imaging results consistent with bvFTD | | | | | | | |
|  |  |  |  |  |  |  |  |  |  | | | C.1. Frontal and/or anterior temporal atrophy on MRI or CT | | | ● | |
|  |  | | | | |  | |  |  | | | C.2. Frontal and/or anterior temporal hypo-perfusion or hypometabolism on PET or SPECT | | | — | |
|  |  | | | | |  | |  |  |  |  |  |  |  |  |  |
|  |  | | | | |  | |  |  | | | | | |  | |
|  |  | | | | |  | |  |  | | | | | |  | |
| bvFTD: behavioral variant frontotemporal dementia; CT: computerized tomography; MRI: magnetic resonance imaging; PET: positron emission tomography; SPECT: single photon emission computerized tomography. | | | | | | | | | | | | | | | | |
|  |  | | | | |  | |  |  | | | | | |  | |
