# Supplementary material for: Case Report: Catatonic Stupor in Behavioral Variant Frontotemporal Dementia
Source: Front Neurol. 2022 Jan 18;12:798264. doi: 10.3389/fneur.2021.798264 (PMC8805594; doi:10.3389/fneur.2021.798264)
Supplement: Supplementary file 2 [file Table_2.docx]

| **Supplementary Table 2. Syndromes of Unresponsive Mutism** | | | |
| --- | --- | --- | --- |
| **Syndrome** | **Phenomenology** | **Pathophysiology** | **Pathological correlates** |
| **Coma** | |  |  |
| A state of acute unresponsiveness in which the eyes remain shut, and postural tone and sleep-wake rhythms are lost (Posner *et al.*, 2019). | | Loss of arousal (phasic activation) and wakefulness (tonic activation). | Lesion or dysfunction of ascending projections from mesopontine tegmentum, paramedian thalamic nuclei, and basal forebrain. |
| **Stupor (neurologic)** | |  |  |
| A state of acute unresponsiveness in which the eyes remain shut, and postural tone and sleep-wake rhythms are lost. It differs from coma because the patient can be momentaneously awakened by sufficiently vigorous external stimulation (Posner *et al.*, 2019). | | Preservation of arousal (phasic activation) with loss of wakefulness (tonic activation). | Lesion or dysfunction of ascending projections from basal forebrain with relative sparing of ascending activating projections from paramedian thalamic nuclei and mesopontine tegmentum. |
| **Unresponsive wakefulness syndrome (former apallic-vegetative state)** | | |  |
| A state of unresponsiveness in which the eyes spontaneously remain open and shut for long periods indicating the alternation of sleep-wake rhythms. If asleep, the patient can be awakened by external stimulation. Both arousal and wakefulness are thus relatively preserved. There are, however, no overt signs of subjective mental activity (Laureys *et al.*, 2010). | | Apallic-vegetative states represent the human analogous of experimental decortication with preservation of subcortical nuclei and white matter; they may also result from cortical inactivation by lesions in strategic basal forebrain and thalamic nuclei. | (i) Massive destruction or dysfunction of the isocortex with sparing of ascending activating projections from mesopontine tegmentum, paramedian thalamic nuclei, and basal forebrain (Ingvar *et al.*, 1978); or bilateral lesions of critical structures that enable cortical function: (ii) the septohypothalamic junction (Jefferson, 1957), and (iii) the medial thalamic nuclei (Edlow *et al.*, 2012). |
| **Akinetic mutism** | |  |  |
| A state of unresponsiveness in which the eyes spontaneously remain open and shut for long periods indicating the alternation of sleep-wake rhythms. In contrast to the apallic-vegetative states, the eyes often follow moving targets, such as the face of relatives and health personnel. | | The extreme end of a spectrum of severity of diminished motivation, which varies from abulia minor to akinetic mutism (Fisher, 1984). | Bilateral lesions falling within the anterior paramedian core of brain (superomedial prefrontal and anterior cingulate cortices) and Nauta’s limbic midbrain area (*d*e Oliveira-Souza *et al.*, 1995; Nauta *&* Domesick 1981); implies sparing of occipito-tegmental projections, which mediate the visual pursuit of moving targets (Dougherty *et al.*, 1981). |
| **Brain in vat syndrome** | |  |  |
| Absence of any evidence of subjective mental life, sleep-wake rhythms, and postural tonus (Damasio, 1994). | | Disconnection of the brain from sensory and motor peripheral organs. | Massive demyelination of sensory and motor cranial and peripheral nerves (Young, 2003). |
| **Mesencephalic locked-in syndrome** | |  |  |
| Mutism, total ophthalmoplegia, and quadriplegia with preservation of conscious awareness, which cannot be expressed because no residual motor channel, such as vertical eye movements, are left unscathed. | | Oculomotor and faciolingual paralysis, and paralysis of trunk and four limbs. | Bilateral destruction of oculomotor nerves and nuclei, corticobulbar and corticospinal tracts in the midbrain (*d*e Oliveira-Souza, 2012). |
| **Stupor (catatonic)** | |  |  |
| A state of unresponsiveness in which the eyes may remain open or shut, usually associated with the adoption of odd postures and stereotypies of gaze, face, and hands (Fisher, 1989). | | Imbalance of the neural systems that mediate the fundamental interactions of the individual with the environment towards the avoidance pole of the approach-avoidance spectrum. | Predominance of prefrontal (avoidance) in relation to parietal (approach) circuits (Vilensky *&* Gilman, 1977). |

**References**

*d*e Oliveira-Souza, R. (2012). The human extrapyramidal system. *Medical Hypotheses*, *79*, 843-852. doi: 10.1016/j.mehy.2012.09.004.

*d*e Oliveira-Souza, R., Bezerra, M. L. S., Figueiredo, W. M., & Andreiuolo, P. A. (1995). Hematoma tegmento-mesencefálico espontâneo. Aspectos neurocomportamentais do terço rostral do tronco encefálico humano. *Arquivos de Neuropsiquiatria, 53,* 807-814. doi: 10.1590/S0004-282X1995000500018.

Damasio, A. R. (1994). *Descartes’ Error: Emotion, Reason and the Human Brain*. New York, NY: Avon Books.

Dougherty, J. H., Rawlinson, D. G., Levy, D. E., & Plum, F. (1981). Hypoxic-ischemic brain injury and the vegetative state: clinical and neuropathologic correlation. *Neurology, 31*, 991-997. doi: 10.1212/wnl.31.8.991.

Edlow, B. L., Takahashi, E., Wu, O., Benner, T., Dai, G., Bu, L., Grant, P. E., Greer, D. M., Greenberg, S. M., Kinney, H. C., & Folkerth, R. D. (2012). Neuroanatomic connectivity of the human ascending arousal system critical to consciousness and its disorders. *Journal of Neuropathology and Experimental Neurology, 71*, 531-546. doi: 10.1097/NEN.0b013e3182588293.

Fisher, C. M. (1984). Abulia minor vs. agitated behavior. *Clinical Neurosurgery*, *31*, 9-31. doi: 10.1093/neurosurgery/31.cn_suppl_1.9.

Fisher, C. M. (1989). “Catatonia” due to disulfiram toxicity. *Archives of Neurology*, *46*, 798-804. doi: 10.1001/archneur.1989.00520430094024.

Ingvar, D. H., Brun, A., Johansson, L., & Samuelsson, S. M. (1978). Survival after severe cerebral anoxia with destruction of the cerebral cortex: the apallic syndrome. *Annals of the New York Academy of Sciences, 315*, 184-214. doi: 10.1111/j.1749-6632.1978.tb50339.x.

Jefferson, G. (1957). The reticular formation and clinical neurology. In: H. H. Jasper, L. Proctor, R. Knighton, & R. T. Costello (Editors) *Reticular Formation of the Brain*. *Henry Ford Hospital, Detroit, International Symposium* (p. 729-738). Churchill.

Laureys, S.,  Celesia, G. G., Cohadon, F., Lavrijsen, J., León-Carrión, J., Sannita, W. G., Sazbon, L.,  Schmutzhard, E.,  von Wild, K. R., Zeman, A., Dolce, G., and the European Task Force on Disorders of Consciousness. (2010). Unresponsive wakefulness syndrome: a new name for the vegetative state or apallic syndrome. *BMC Medicine*, *8*, 68. doi: 10.1186/1741-7015-8-68.

Nauta, W. J. H., & Domesick, V. B. (1981). Ramifications of the limbic system. In: S. Matthysse (Editor) *Psychiatry and the Biology of the Human Brain: A Symposium Dedicated to Seymour S Kety*. New York, NY: Elsevier-North Holland, p. 165-188.

Posner, J., Saper, C. B., Schiff, N. D., & Claassen, J. (2019). *Plum and Posner’s Diagnosis and Treatment of Stupor and Coma, fifth edition*. Oxford, UK: Oxford University Press.

Vilensky, J. A., & Gilman, S. (1977). Positive and negative factors in movement control: A current review of Denny-Brown’s hypothesis. *Journal of Neurological Sciences*, *151*, 149-158. doi: 10.1016/S0022-510X(97)00134-2.

Young, G. B. (2003). De-efferentaion and de-afferentation in fulminant polyneuropathy: Lessons from the isolated brain. *Canadian Journal of Neurological Sciences*, *30*, 305-306. doi: 10.1017/s0317167100002997.
